# Predicting current habitat suitability for intermediate snail hosts of urogenital and intestinal schistosomiasis in the Lower Shire Valley floodplain of southern Malawi

**DOI:** 10.1186/s13071-025-06952-3

**Published:** 2025-08-29

**Authors:** Clinton Nkolokosa, James Chirombo, Christopher M. Jones, Rex B. Mbewe, Eggrey Aisha Kambewa, Peter Makaula, Julie-Anne Akiko Tangena, J. Russell Stothard

**Affiliations:** 1 Malawi-Liverpool-Wellcome Programme, Blantyre, Malawi; 2 Liverpool School of Tropical Medicine, Liverpool, L3 5QA UK; 3 Kamuzu University of Health Sciences, Malaria Alert Centre, Blantyre, Malawi; 4 University of Stirling, Stirling, FK9 4LA Scotland, UK

**Keywords:** Species distribution modelling, Ensemble machine learning, Schistosomiasis, *Bulinus africanus* group, *Biomphalaria pfeifferi*, Malawi, Climate change

## Abstract

**Background:**

Relating the geographical distribution of intermediate freshwater snail hosts (viz. vectors of schistosomes) to local environmental attributes offers value for understanding the epidemiological landscape of schistosomiasis transmission in a changing aquatic environment. Schistosomiasis—both urogenital and intestinal—causes significant human suffering, affecting approximately 240 million people globally and grouped within the neglected tropical disease (NTD) umbrella. This study addresses the following questions: 1. Where are the most suitable habitats for intermediate host snails in the Lower Shire Valley (LSV) in Malawi? 2. Which environmental factors are strongly associated with the geographical distribution of such snails in the LSV?

**Methods:**

This paper presents the first species distribution models (SDMs) for intermediate snail hosts for urogenital and intestinal schistosomiasis in Chikwawa and Nsanje Districts, which together form the LSV). The SDMs developed for this study are ensemble machine learning approaches based on Random Forest (RF), Support Vector Machines (SVM), and multilayer perceptron (MLP) and are specific to the *Bulinus africanus* group and *Biomphalaria pfeifferi*. The former transmits urogenital schistosomiasis (*Schistosoma haematobium*), while the latter transmits intestinal schistosomiasis (*Schistosoma mansoni*).

**Results:**

The SDMs reveal the following: 1) currently, *Bu. africanus* group not only has a wide distribution across central Chikwawa and eastern Nsanje but is also concentrated in floodplains, and the LSV has few habitats that can support *Bi. pfeifferi*, and 2) vegetation cover is the most important predictor of *Bu. africanus* group distribution, whereas precipitation variables are most important for *Bi. pfeifferi* in the LSV. Thus, *Bu. africanus* group habitat is the most dominant and abundant, while *Bi. pfeifferi* suitable habitat is patchy and scarce.

**Conclusion:**

The distribution of suitable habitats for potential urogenital and intestinal schistosomiasis transmission across LSV is not uniform and typically non-overlapping. Understanding the spatial and temporal distributions of these snails is important for controlling and eliminating schistosomiasis.

**Graphical Abstract:**

**Figure:**
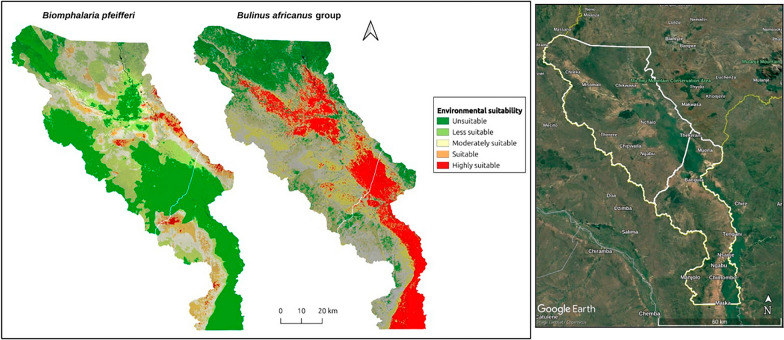

**Supplementary Information:**

The online version contains supplementary material available at 10.1186/s13071-025-06952-3.

## Background

Relating the geographical distribution of intermediate freshwater snail hosts (viz. vectors of schistosomes) to local environmental attributes offers value for understanding the epidemiological landscape of schistosomiasis transmission in a changing aquatic environment. Schistosomiasis—both urogenital and intestinal forms—causes significant human suffering, affecting approximately 240 million people globally and grouped under the neglected tropical disease (NTD) umbrella. The disease is endemic in Malawi. Approximately 40–50% of the Malawi population are at risk of being infected, with school-aged children showing the highest infection rates and constituting the most affected group [1–3]. If schistosomiasis is not treated, it can result in severe health complications, including infertility, anemia, malnutrition, abdominal pain, enlarged or damaged liver, hematuria, and blood in the stool [3, 4]. Stigma, social exclusion, and poor educational outcomes aggravate the suffering from schistosomiasis [3]. Schistosomiasis is caused by parasitic flatworms and is strongly associated with human water contact behavior [3]. The parasitic worms (trematodes) that cause the disease are of the genus *Schistosoma*, with *Schistosoma haematobium* and *Schistosoma mansoni* being the most important species in Malawi [5, 6]. The latter is responsible for intestinal schistosomiasis, whereas the former causes urogenital schistosomiasis. The transmission chain depends on compatible snails. In Malawi, *S. mansoni* and *S. haematobium* parasites are transmitted by freshwater-intermediate host snails of the genera *Biomphalaria* (Planorbidae) and *Bulinus* (Planorbidae), respectively [5, 7]. Larval schistosomes (cercariae) from infected freshwater snails penetrate human skin, causing (re)infection. This occurs in water bodies, for example, during routine agricultural, domestic, occupational and recreational activities, such as irrigating, washing, wading, bathing, or swimming. Therefore, human–surface water interactions and the presence of intermediate host snails predicate the spatial distribution of schistosomiasis prevalence. This is particularized in Malawi, where the consequence of proximity to surface water, such as lakes, rivers, wetlands, canals, dams, or ponds—a generally high risk—is the prevalence of schistosomiasis [1, 5, 7–11]. Located in southern Malawi and comprising two districts—Chikwawa and Nsanje—the Lower Shire Valley (LSV) is the quintessence of high schistosomiasis burden in marginalized communities that rely heavily on surface water for their livelihoods (agriculture and fishing) or daily living (bathing, washing, cooking, and drinking). Here, epidemiological surveys highlight a noticeable schistosomiasis burden. For example, in the 1980s Teesdale and Chitsulo [12] reported *S. haematobium* infection rates of 47% in 1977, which dropped to 31% 5 years later in 1982. Then, 39 years later, Chiepa et al. (2) reported an overall prevalence of 35.0% (95% confidence interval [CI] 33.6–36.5) for egg-patent urogenital schistosomiasis across 21 primary schools in Chikwawa, and through focalization, prevalence was in excess of 50.0% in certain schools. At the same time, a contemporary malacological survey in 2023 confirmed the presence of *Biomphalaria* habitats in the LSV [13]. Therefore, the LSV provides a unique geographical context for elucidating the geographical suitability to schistosomiasis, environmental characteristics of snail habitats, and distribution—all of which have not been fully explored in this study area.

Among the different approaches used to control the spread of schistosomiasis, snail control is essential for interrupting the parasite life cycle [6]. Eliminating snail hosts is considered an important, effective, and convenient strategy for schistosomiasis prevention in endemic areas [14, 15]. Accordingly, the WHO recommends prevention and treatment: molluscicidal (chemical) control, ecological control (sanitation and environmental modification), and health education for behavioral change [16]. Identifying the habitats where intermediate host snails occur could actively inform NTD control programs to address schistosomiasis, which is evidently an important public health challenge in LSV, southern Malawi [1, 17]. The Malawi Neglected Tropical Diseases Master Plan (2023–2030) recognizes that a changing climate is a threat and challenge to schistosomiasis control [18]. However, in the absence of evidence, endemicity dynamics resulting from anthropogenic, climatic and ecological changes remain unclear. Revealing the current and future schistosomiasis risk is vital for opportune control efforts and resolving the elusive climate change challenges, holding back national and global efforts to eliminate NTDs in resource-constrained settings, such as Malawi.

To date, several studies across Africa have investigated the abundance, distribution, and spread of freshwater snails at broad scales via a species distribution model (SDM). The task of an SDM is to determine the probability of a species occurring in a particular habitat as a function of a set of environmental conditions. For example, in Kenya, potential habitats of freshwater snails were mapped via an SDM on the basis of maximum entropy (Maxent) [19]. This study incorporated a set of environmental variables, including land surface temperature, soil pH, and vegetation greenness, to predict environmental suitability [19]. In a related study, Maxent was also applied to forecast the distribution of suitable habitats for *Bu. globosus* and *Bi. pfeifferi* in South Africa [20]. In Malawi, Reed et al. [10] provided a first step toward understanding the spatial risk of intestinal and urogenital schistosomiasis within sampled areas via two-dimensional (2D) mean Gaussian process prediction. In the present study, machine learning (ML) classifiers (random forest [RF], support vector machine [SVM], and multilayer perceptron [MLP]) are utilized in potential distribution modelling over a mosaic habitat. The benefit of this approach is that ML methods excel at detecting patterns in complex environmental data, including nonlinear interactions between species and their environment. Temperature, precipitation, geographical elevation, terrain slope, soil type, distance to a water body, and normalized difference vegetation index (NDVI) variables were used as predictors. Beyond LSV, this study provides an important opportunity to advance the understanding of geographic distribution of freshwater snail habitat suitability attributed to environmental conditions. Thus, the importance of this study is by no means restricted to intestinal and urogenital schistosomiasis in LSV but also potentially to other NTDs and forms of schistosomiasis of veterinary importance in other geographical settings. Therefore, with respect to LSV, the aim of this study is twofold: (1) developing and parameterizing an SDM for *Bu. africanus* group and *Bi. pfeifferi* snails in LSV, and (2) using the parameterized model to nowcast the distribution of the *Bu. africanus* group and *Bi. pfeifferi* in LSV under current (2023) climatic and environmental conditions.

## Methods

### Study area and species occurrence data

The LSV study area (latitude: 14°25′ S and 16°55′ S and longitude: 35°16′ E and 35°12′ E) is the southern subregion of Malawi, which is situated in the lower Shire floodplain and consists of two districts: Chikwawa and Nsanje. The study area, covering approximately 6833 km^2^, is characterized by a wide range of topography (medium-altitude plain 750–1300 m and floodplain 35–105 m) and ecological zones (freshwater habitats, tropical grasslands, savannas and shrublands, montane forest–grassland mosaics, and flooded grasslands) [21, 22]. The climate is subtropical: a warm-wet season from November to April and a hot-dry season from May to October [21]. According to the National Statistics Office, Chikwawa and Nsanje had estimated population densities of 128 people/km^2^ and 168 people/km^2^ in 2023, respectively [23]. Across the valley, schistosomiasis is endemic and focal, with district-level prevalence considered low for *S. mansoni* and moderate for *S. haematobium* [18]. For example, in 2017, the schistosomiasis prevalence rates in Chikwawa and Nsanje were 15.8% (95% CI 10.9, 22.4) and 25.4% (95% CI 15.3, 38.9), respectively [18].

Data of *Bu. africanus* group and *Bi. pfeifferi* occurrence (absence/presence) were collected in the floodplains of the LSV between May and June 2023. This study surveyed a total of 70 sampling sites across the LSV: Chikwawa (*n* = 35) and Nsanje (*n* = 35). The inclusion criteria were as follows: (1) confirmed cases of intestinal or urogenital schistosomiasis in the area and (2) the presence of surface water (wetlands, rivers, dams, ponds, canals), including sites previously reported by Nkolokosa et al. [13]. Here, a malacologist and three trained field collectors who adhered to the World Health Organization (WHO) sampling design protocol [16] collected the survey data and used a field guide for African freshwater snails to identify the snail intermediate hosts [24, 25]. Standardized sampling effort was achieved by setting the sampling time to 15 min per site. The collected data set includes information on the geographic coordinates of the sampled sites, site number, date and time of collection, all freshwater snails encountered (*Lymanea*, *Melanoides*, and *Lanistes*), habitat type, human– and animal–water contact, aerial photograph of the site, and geographical elevation. However, examining these additional data is outside the scope of this study. A hand-held global positioning system (GPS) device (Garmin Montana 700 GPS, US) was used to locate and map the sites. Typical of studies determining the distribution of freshwater snails, field sampling was designed on the basis of the knowledge that such snails occur in lentic and lotic ecosystems: standing water and running water habitats [10, 26–28]. Therefore, the survey targeted representative aquatic habitats, such as lakes and marshlands, ponds and pools, and rivers and canals, especially where human–water contact occurs (fishing, gardening, bathing, swimming, washing, river crossing, sand harvesting, making mudbrick, etc.).

### Environmental predictors

The present study uses abiotic (climatic, topographic, pedologic [soil], and proximity to water) and biotic (vegetation cover) factors to determine the association of such environmental factors and gradients on *Bu. africanus* group and *Bi. pfeifferi* distributions (Additional file: Table 1). Previous studies have shown that abiotic factors, such as rainfall [29, 30], air temperature [29, 31], and altitude [32, 33], influence the distribution and habitat preference of freshwater snails of medical importance. For example, it has been demonstrated that temperature affects the mortality, fecundity, and growth rate of *Bu. africanus* group and *Bi. pfeifferi* snails [30, 34], and precipitation and elevation gradients are negatively correlated with freshwater snail distribution and abundance [33, 35–37]. The velocity of water tends to have a positive relationship with the slope gradient [38, 39]. Generally, *Bulinus* and *Biomphalaria* snails prefer stagnant or slow flow (velocity ≤ 0.3 m/s) [40, 41]. The argument is that rapidly flowing water impedes the establishment of reproductive colonies and displaces the snail population [30]. This study uses slope as a direct factor regulating water velocity, which is a key determinant of intermediate host snail habitat preference. However, the effect of slope on the distribution of the *Bu. africanus* group and *Bi. pfeifferi* across the LSV has yet to be determined.

The distance to water or surface water proximity variable was included because the distribution of freshwater snails is determined by surface water (permanent or ephemeral). In accordance with previous studies, this study posits that surface water influences the composition of habitat variables relevant to the *Bu. africanus* group and *Bi. pfeifferi*. For example, vegetation composition and soil characteristics promote or inhibit (micro)biological processes [30, 35, 42, 43]. Notably, typically *Bu. africanus* group and *Bi. pfeifferi* snails are frequently found close to water‒land edges [10]. Hence, the presence of and proximity to a waterbody were predicted to be positively associated with *Bu. africanus* group and *Bi. pfeifferi* presence. It was also expected that soil properties would have an intermediate and interactive causal influence on *Bu. africanus* group and *Bi. pfeifferi* occurrence, resulting in spatial associations of particular soil group(s) with the presence or absence of snails [10]. This is because several lines of evidence show that soil properties—physical (texture, porosity, color) and chemical (mineralogy, organic matter content, acidity, and alkalinity)—significantly govern soil water dynamics, ultimately affecting plant assemblages and the population growth of intermediate snail hosts [44, 45].

The bioclimatic data (19 Bioclim variables) were retrieved from the WorldClim database (https://www.worldclim.org/data/bioclim.html), see Supplementary Table 1. The Bioclim variables were downloaded using the R package geodata (version 0.6–2) [46]. The elevation and soil group data sets were obtained from the Malawi Spatial Data Platform (MASDAP, http://www.masdap.mw/). On MASDAP, the soil data are available as a vector and were converted to a categorical raster using the Rasterize tool in QGIS 3.22.1 [47]. On the basis of the national Soil and Terrain database for Malawi, a total of ten soil groups were identified for the study area: Luvisols, Arenosols, Cambisols, Solonetz, Gleysols, Leptosols, Lixisols, Phaeozems, Solonetz, and Vertisols. The slope (Shuttle Radar Topography Mission-based) and distance to water bodies (OpenStreetMap-based) data were obtained from WorldPop (https://hub.worldpop.org/project/categories?id=14). The NDVI, which is a quantitative measure of vegetation greenness, was computed from Sentinel-2 imagery in Google Earth Engine [48]. This study aggregated the seasonal NVDI time series for 2022–2023 (May–June) to derive the mean annual NDVI. The purpose of this was to identify how vegetation density and changes in greenness, as captured in a satellite image, affect the distribution of the *Bu. africanus* group and *Bi. pfeifferi* in LSV. This is because the removal or establishment of vegetation cover, particularly invasive macrophytes and hydrophytes, alters snail abundance and human schistosome transmission. For example, hydrophyte cover by water hyacinth is highly correlated with snail abundance and increases the total production of human-infectious cercariae sixfold [49].

### Species distribution modelling

To predict the suitable habitat areas for the *Bu. africanus* group and *Bi. pfeifferi* using a suite of environmental covariates, an SDM model was developed on the basis of the ensemble (consensus) method comprising the following ML models: RF, SVM and MLP (Table 1) [50, 51]. Using the ensemble and sdm functions from the *sdm* package (version 1.2–56) in R Statistical Software (version 4.2.2) [52], RF, SVM, and MLP—nonparametric ML models—were used to classify the presence or absence of the *Bu. africanus* group and *Bi. pfeifferi* without making assumptions about the occurrence data [50]. RF is an ensemble learning method that constructs multiple decision trees to enhance predictive performance and reduce overfitting [53]. SVM is a classification method that finds the hyperplane that best separates the data into different classes [54]. MLP is an artificial neural network method that is composed of multiple layers of interconnected nodes, each performing a weighted sum of inputs followed by a nonlinear activation function [55]. The models were trained using fivefold crossvalidation and bootstrap partitioning. Each crossvalidation had 10 runs, resulting in 60 runs per model and 180 in total, with all other settings left as default. The RF model was configured with 1000 trees and three variables tried at each split. For the MLP, a maximum of 500 iterations was set, using a random weight initialization and standard back-propagation with a learning rate of 0.2. The SVM model, implemented using a Gaussian Radial Basis Function (RBF) kernel, utilized a cost parameter (C) of 1 and a sigma value of 0.1149.

**Table 1:** Fitted snail SDMs

Model	Description	Definition	
RF	Ensemble of decision trees with randomization		Classification: ${\hat{C} }_{rf}^{B}\left(x\right)=majorityvote{\{{\hat{C} }_{b}(x)\}}_{1}^{B}$ Where: ${\hat{C} }_{rf}^{B}\left(x\right)$ is the predicted class for an input $x$ based on RF, ${\hat{C} }_{b}(x)$ is the predicted class for $x$ from the $bth$ tree in the forest, $B$ is the total number of trees in the forest, and $majorityvote$ is the class that appears most frequently among the predictions [53]
SVM	Finds optimal separation hyperplane in feature space		Classification rule: $h\left(x\right)={\sum }_{i=1}^{n}{\alpha }_{i}{y}_{i}K\left({X}_{i},X\right)+b$ Where: ${\alpha }_{i}$ is the Lagrange multiplier found during optimization, ${y}_{i}$ are class labels (+1 or −1), $K\left({X}_{i},X\right)$ is the Kernel function measuring similarity between data points, $n$ is the number of support vectors, and $b$ is the bias term (offset) that shifts the decision boundary [54]
MLP	A type of neural network composed of multiple layers of nodes		Prediction: $\hat{y}\left( x \right)\, = \,f\left( {W^{\left( L \right)} \, \cdot \,f\left( {W^{{\left( {L - 1} \right)}} \cdot \ldots \,f\left( {W^{\left( 1 \right)} \, \cdot \,x\, + \,b^{\left( 1 \right)} \,} \right) + \,b^{{\left( {L - 1} \right)}} \,} \right)\, + b^{\left( L \right)} } \right)$ Where: ŷ($x$) is the predicted output of the MLP as a function of the input $x$, in this case class label (presence or absence), $f(\bullet )$ is the activation function applied element-wise at each layer, $L$ is the total number of layers in the network (excluding the input layer), $x$ is the input vector (features of the data), ${W}^{(i)}$ is the weight matrix for the $i$-th layer, ${b}^{(i)}$ is the bias vector for the $i$-th layer, and $\cdot$ is the matrix multiplication between layers [55]

There is growing recognition of the importance of combining models, often referred to as ensemble modelling, with the aim of capturing complementary strengths from individual algorithms. This approach enhances predictive performance, robustness, and generalizability by leveraging diverse model architectures and perspectives [50]. Thus, this study employed weighted model averaging to integrate predictions from three fitted models: RF, SVM, and MLP. Here, the predictions were combined using weighted averaging, with weights assigned on the basis of predictive performance measured by the area under the curve (AUC) statistic from the crossvalidation. The ensemble raster predictions were visualized using QGIS (version 3.34).

Potential multicollinearity (nonindependence of predictor variables) was evaluated by using two specialized R packages: *sdm* and *usdm* (version 2.1–7) [56]. First, variance inflation factors (VIFs) were calculated using the *usdm* package. The VIF is a measure used to detect the severity of multicollinearity. A VIF value above 10 indicates high collinearity; however, a threshold of 5 is not uncommon [56]. Subsequently, the vifstep function available from the *sdm* package was used to detect and exclude variables with the highest VIF (> 10 threshold). The collinearity test is particularly useful in the SDM because, if the predictor variables are highly correlated, the individual effect of each variable is difficult to delineate. A major problem with this approach is that it can cause inflation of uncertainty in parameter estimates and unstable variable importance scores. Consequently, erroneous extrapolation and wrong identification of relevant variables in a model can occur.

### Model performance evaluation

Several performance metrics were applied to assess the classification accuracy and robustness of the models presented in the study. The receiver operating characteristic (ROC) area under the curve (AUC) was used as the primary metric for measuring a model’s discrimination ability. This statistic evaluates the trade-off between sensitivity (the proportion of correctly predicted presences) and specificity (the proportion of correctly predicted absences) [50, 57]. An AUC score of 1 represents perfect classification, whereas a score of 0.5 suggests that the model performs no better than random classification. In addition to the ROC-AUC, the true skill statistic (TSS) and correlation coefficient (COR) were also calculated to provide a more comprehensive assessment of model performance. Here, COR is used to evaluate the strength and direction of the relationship between observed values (true outcomes) and predicted values (model predictions) [56], with a high positive COR (close to 1) indicating strong alignment and good performance, a COR near 0 indicating poor performance, and a negative COR indicating inverse correlation and poor performance. The TSS is a balanced measure of both sensitivity and specificity [57]. The TSS values range from −1 to 1, with values closer to 1 indicating better model performance. A TSS value of 0 or less indicates that a model’s predictive capacity is no better than random. By applying the ROC-AUC, COR, and TSS, the present study ensured a robust evaluation of the models’ ability to accurately predict *Bi. pfeifferi* and *Bu. africanus* snail distribution on the basis of environmental variables. The ROC curve was generated using the roc function in the *sdm* package.

### Relative variable importance

This analysis evaluated the relative importance of each environmental variable included in the ensemble of predictive models. This study implemented the permutation-based variable importance available in the *sdm* package using the getVarImp function [50]. The purpose of this was to identify the most important variable(s) determining the geographic distribution of the *Bu. africanus* group and *Bi. pfeifferi* in the LSV floodplain. Two complementary evaluation metrics, AUC and COR (Pearson correlation) were used to quantify the contribution of each variable. This dual evaluation allowed for a more comprehensive understanding of each variable’s contribution to the model’s predictive performance. The variable importance plot was visualized using *ggpubr* package (version 0.6.0) in R to create a publication-ready figure [58].

## Results and discussion

### Observed distribution of *Bu. africanus *group and *Bi. pfeifferi* in LSV

**Fig. 1:**
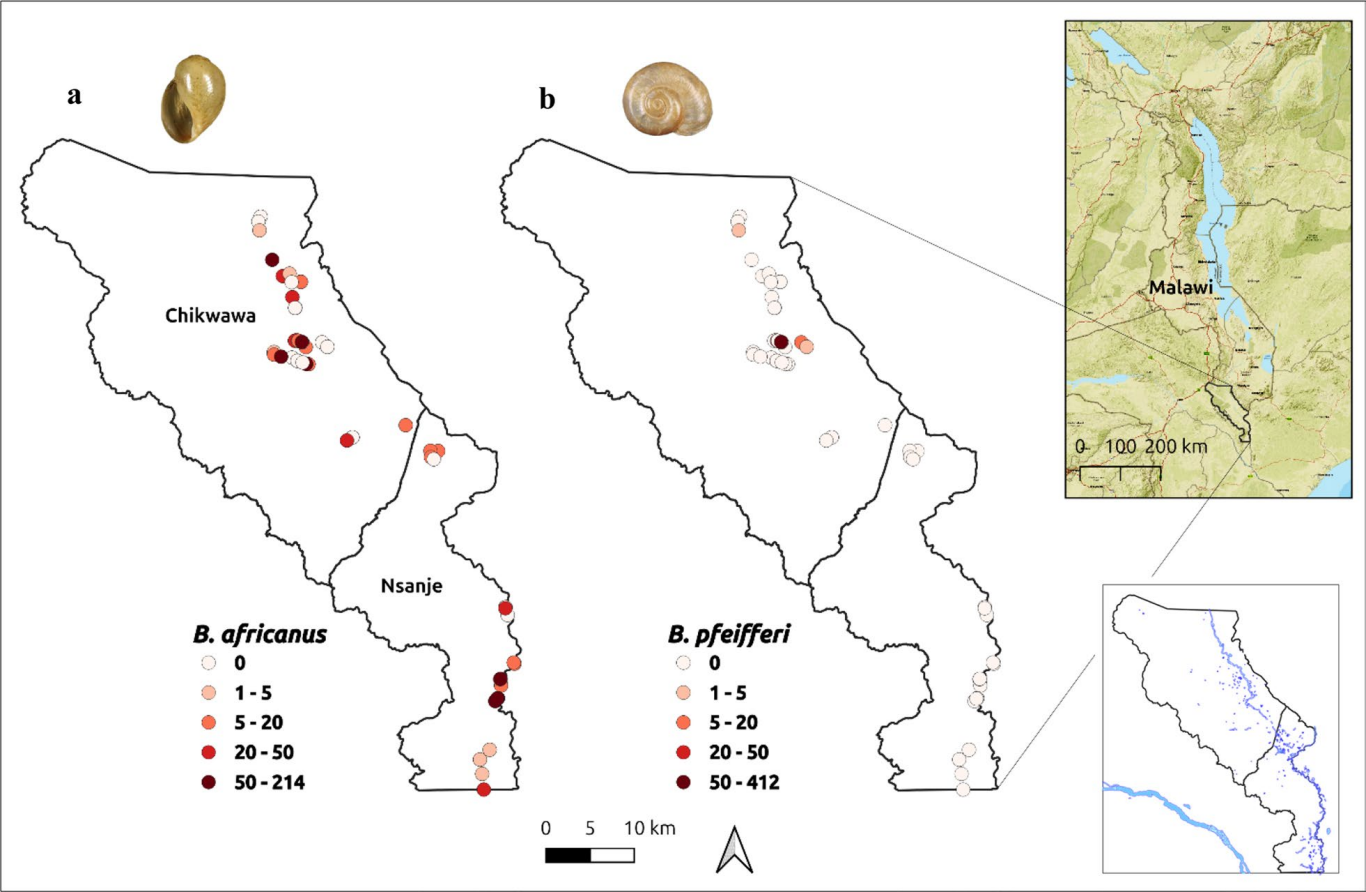
Study area: the sampled locations and numerical abundances of the (**a**) *Bu. africanus* group and (**b**) *Bi. pfeifferi* in Chikwawa and Nsanje during the 2023 malacological survey mapped using QGIS. The insert maps show permanent water bodies and the geographical location of LSV in the context of Malawi

From the malacological surveys, georeferenced occurrence records of the *Bu. africanus* group and *Bi. pfeifferi* were generated along the Shire River, Elephant Marsh, and irrigation canals by foot, car, and boat (Fig. 1). From the survey, a total of 1994 *Bu. africanus* group and 597 *Bi. pfeifferi* snails were collected from 26 and 4 sites, respectively. Of note, no *Bi. pfeifferi* was found in Nsanje.

### Predicted current distribution of the *Bu. africanus* group and *Bi. pfeifferi* in LSV

**Fig. 2:**
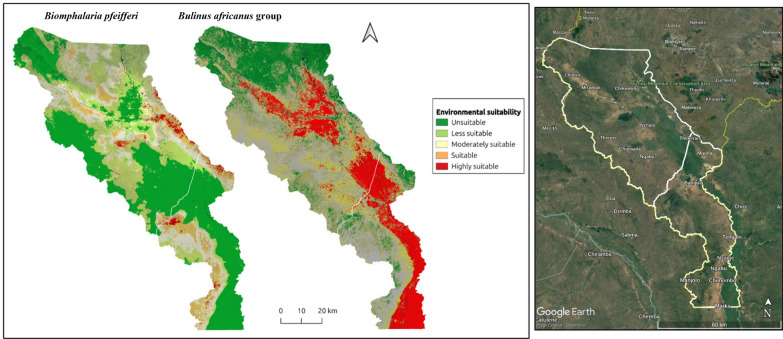
Predicted current (2023) environmental suitability for intestinal and urogenital schistosomiasis transmission in LSV mapped using QGIS 3.34, at a spatial resolution of approximately 144 m. Warmer colors (red and orange) indicate very high to high habitat suitability, whereas green to light green colors indicate very low to low habitat suitability. The suitability was determined using a threshold based on the midpoint between the predicted probability for presence and absence, which in this case corresponded to approximately 0.5. In QGIS, the probability raster was reclassified such that values ≥ 0.5 indicated high suitability and values ≤ 0.5 low suitability. For finer interpretation, suitability was further categorized as follows: ≥ 0.75 = highly suitable and ≤ 0.25 = not suitable

Using an ensemble of RF, SVM, and MLP models, the distributions of the *Bu. africanus* group and *Bi. pfeifferi* in LSV was predicted (Fig. 2). The resulting prediction maps reveal marked differences in range and habitat suitability between the snails. The ensemble model for *Bi. pfeifferi* showed that a substantial area in LSV were an unsuitable habitat for *Bi. pfeifferi*. Broadly, contemporary environmental conditions appear harsh for the snail. However, at the fine scale, Chikwawa contains pockets of suitable conditions. As a result, a few areas in Chikwawa—namely Nchalo, Chipwaila, and Ngabu—are identified as suitable habitats for *Bi. pfeifferi*. Similarly, in Nsanje, greater suitability was predicted in a few areas around Manjolo, Ngabu, and bordering Chikwawa South. Thus, in both districts, the majority of areas were characterized by a very low to low probability of presence. Furthermore, moderate suitability—albeit scanty—was nowcast along the Mwanza River near Misomali, Nchalo and Bangula.

For the *Bu. africanus* group, the ensemble model shows that in most areas of Chikwawa and Nsanje, particularly along the Mwanza River, Shire River, and marshland, the environment is suitable for this host. The map illustrates that the areas highly suitable for *Bu. africanus* are present in riverine and wetland areas. This is particularly evident in floodplain areas with inundated vegetation and vegetation cover, which presented the widest distributions of snails in both districts. The *Bu. africanus* distribution map also indicates a large ecologically unsuitable habitat in northern Chikwawa and a small unsuitable habitat in western Nsanje. Epidemiological surveys in 2024 indicate a high prevalence of urogenital schistosomiasis among school-aged children in Chikwawa (35.0%, 95% CI 33.6–36.5), and snail surveys revealed a wide distribution and high abundance of *Bulinus*. In contrast, intestinal schistosomiasis prevalence was low (1.9%, 95% CI 1.4–2.3), likely attributable to the rarity and restricted range of *Bi. pfeifferi* [13, 59]. This study detected *Bi. pfeifferi* at only 4 out of 70 sites, indicating that it is a rare species with few positive observations (Fig. 1a). This suggests that, while the study area can be considered low risk for intestinal schistosomiasis, the disease remains highly focalized. In summary, these results show that the modeled distribution of the *Bu. africanus* group shows large areas of highly suitable habitat in central Chikwawa and eastern Nsanje. In contrast, the predicted distributions of *Bi*. *pfeifferi* shows a constrained range and few suitable habitats that can support the snail (Supplementary Fig. 3). Interestingly, the marshlands appear to be completely unsuitable habitats for *Bi*. *pfeifferi*. The most suitable habitats for *Bi. pfeifferi* are sparse and fragmented and do not overlap with those of the *Bu. africanus* group. These findings reinforce the idea that *Bulinus* host snails are generalist species with higher survival rates and broad tolerances to varying environmental conditions (e.g., [60, 61] than *Biomphalaria* in the bioclimatic areas of Africa.

### Multicollinearity

Among the 24 input variables, nine variables showed acceptable levels of multicollinearity, as indicated by their VIF values, all of which are below the threshold of 10 (Table 2). This indicated a well-balanced set of variables with negligible multicollinearity. The minimum linear correlation coefficient was 0.004 between bio15 and bio6. This indicates a very weak positive correlation between these two variables. The maximum linear correlation coefficient was 0.822 between bio15 and bio13, indicating a strong positive correlation between these two variables.

**Table 2:** VIFs of the variables retained in the ensemble model

Variable	VIF	Description
bio 1	3.67	Mean annual air temperature
bio 3	5.09	Isothermality: the ratio of the diurnal variation to the annual temperature range
bio 6	4.43	Minimum temperature of coldest month
bio 13	9.19	Precipitation amount of wettest month
bio 15	5.27	Precipitation seasonality
Slope	2.54	Topographic slope
Soil	2.18	Dominant soil group based on FAO classification
Dist_water	1.61	Distance to the nearest water body
NDVI	1.79	Normalized difference vegetation index

For the complete description of each variable, please refer to the data source. FAO, Food and Agriculture Organization

### Relative variable importance

**Fig. 3:**
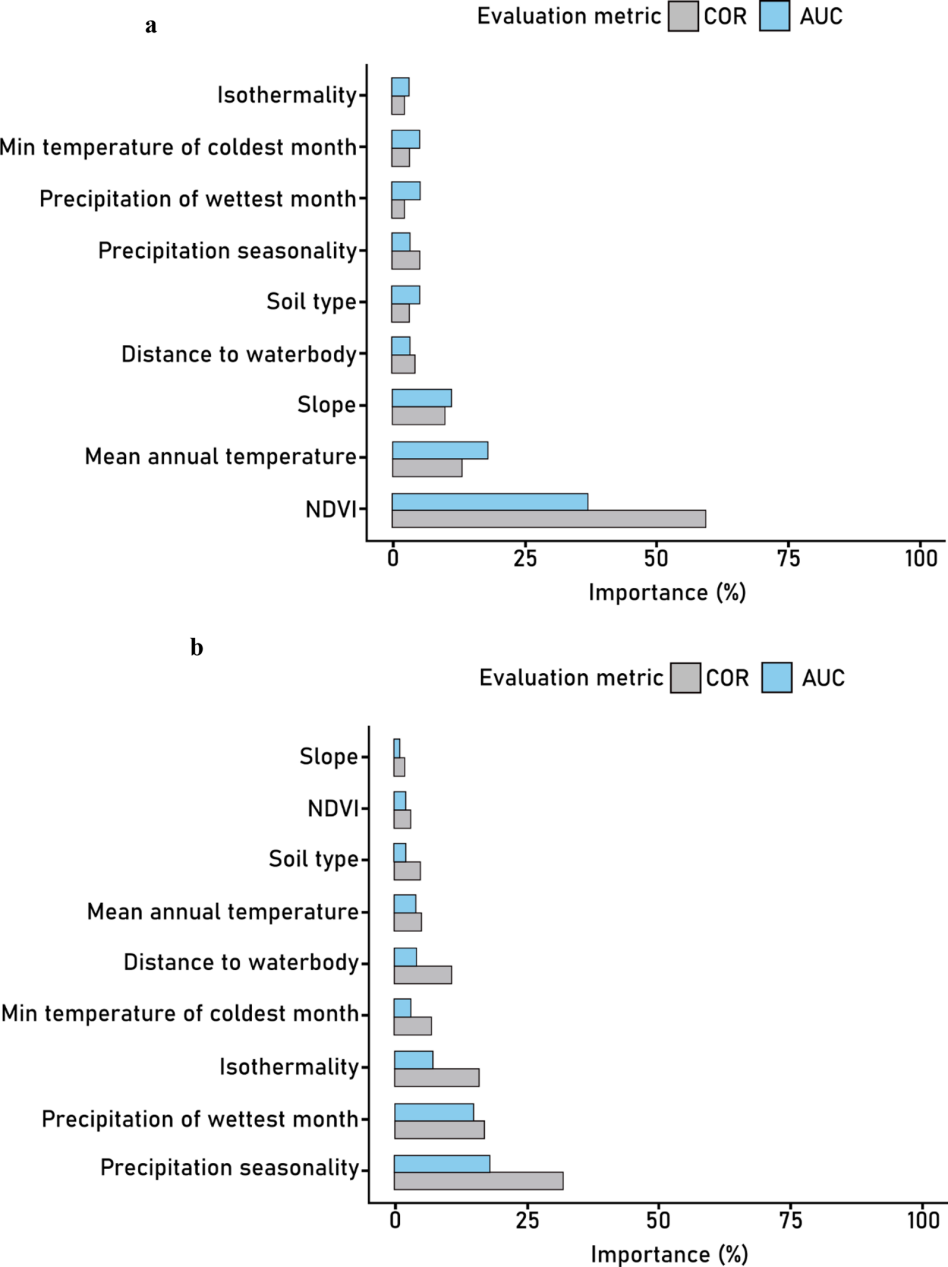
Variable importance plots for **a** *Bu. africanus* group and **b** *Bi. pfeifferi* ensemble models

The variable importance plot for *Bu. africanus* ensemble (Fig. 3a) shows that the most important variable in the model is the NDVI (COR = 0.59, AUC = 0.37). The next two important variables are the mean annual temperature (bio1, COR = 0.13, AUC = 0.18) and slope (COR = 0.09, AUC = 0.11). The other variables had lower importance: distance to the water body (COR = 0.04, AUC = 0.03), soil type (COR = 0.03, AUC = 0.05), precipitation seasonality (bio15, COR = 0.05, AUC = 0.03), precipitation in the wettest month (bio13, COR = 0.02, AUC = 0.05), minimum temperature in the coldest month (bio6, COR = 0.03, AUC = 0.05), and isothermality (bio3, COR = 0.02, AUC = 0.03).

For *Bi. pfeifferi* (Fig. 3b), precipitation seasonality (COR = 0.32, AUC = 0.18) was the most important variable, followed by precipitation in the wettest month (COR = 0.17, AUC = 0.15) and isothermality (COR = 0.16, AUC = 0.07). The least important variables in order of magnitude from relatively low importance to very low importance are the minimum temperature of the coldest month (COR = 0.07, AUC = 0.03), the distance to the waterbody (COR = 0.11, AUC = 0.04), the mean annual temperature (COR = 0.05, AUC = 0.04), the soil type (COR = 0.05, AUC = 0.02), the NDVI (COR = 0.03, AUC = 0.02), and the slope (COR = 0.02, AUC = 0.01). Although broadly consistent with Monde et al. [62], this outcome is contrary to that of Ponpetch et al. [44], who reported that soil properties are key factors in the distribution of *Bi. pfeifferi* in Ethiopia.

Taken together, it is evident that the explanatory variables had a combinatory effect. Hence, it could conceivably be hypothesized that adding more explanatory variables—such as vegetation and surface water anomalies (droughts and floods)—produces a better picture of fine-scale *Biomphalaria* and *Bulinus* distribution across LSV.

### Study limitation

This study has methodological limitations due to the difficulty in obtaining spatial coverage for proximal environmental predictors, such as physicochemical water parameters (e.g., pH and conductivity). Here, ecological processes (e.g., predation by fish and birds, competition from other aquatic snails, and dispersal barriers such as rapids and mountain ranges), anthropogenic changes (e.g., agricultural expansion), and disturbances (e.g., floods and droughts) were not included in the SDM. Therefore, this study acknowledges the potential influence of other critical environmental predictors that remain unidentified and excluded. For example, the LSV is an agro-landscape currently undergoing large-scale agricultural expansion driven by the Shire Valley Transformation Project. In this geographical context, the expansion of cropland has been shown to significantly alter natural habitats, leading to habitat loss and fragmentation [63]. The loss of habitat and biodiversity can subsequently trigger cascading effects within ecological communities, impacting species abundance, distribution, and interactions [64]. Unlike environmental conditions, biotic processes are rarely integrated into species distribution models, underscoring the need for further attention [65]. In addition, with a few presence records for *Bi. pfeifferi*, caution must be applied, as it is possible that the predictions were mostly influenced by absence records and a moderate sampling effort.

### Model performance and evaluation

Table 3 provides a performance summary of the predictive models used to predict the distributions of the *Bu. africanus* group and *Bi. pfeifferi* in the LSV. The AUC, COR, and TSS values underscore the model’s capacity to delineate suitable snail habitats from unsuitable areas. For *Bu. africanus*, the SVM model achieved the highest performance (AUC = 0.74, COR = 0.51, TSS = 0.56, deviance = 0.85), followed by RF (AUC = 0.73, COR = 0.33, TSS = 0.57, deviance = 0.97), both of which demonstrated high prediction accuracy. In contrast, the MLP showed weak performance (AUC = 0.56, COR = 0.07, and TSS = 0.44) and the highest deviance (2.04), indicating high prediction error. This suggests that the MLP had poor classification performance for *Bu. africanus*, a species that is widely distributed and abundant in the LSV.

**Table 3:** Model performance of *Bu. africanus* group and *Bi. pfeifferi* distribution models across ML model types on the test data set generated using partition

Species	Model type	AUC	COR	TSS	Deviance
*Bu. africanus*	RF	0.73	0.33	0.57	0.97
	SVM	0.74	0.51	0.56	0.85
	MLP	0.56	0.07	0.44	2.04
*Bi. pfeifferi*	RF	0.77	0.34	0.69	0.77
	SVM	0.73	0.38	0.64	0.78
	MLP	0.83	0.42	0.75	0.81

For *Bi. pfeifferi* model, MLP (AUC = 0.83, COR = 0.42, TSS = 0.75, deviance = 0.81), outperformed RF (AUC = 0.77, COR = 0.34, TSS = 0.69, deviance = 0.77), and SVM (AUC = 0.73, COR = 0.38, TSS = 0.64, deviance = 0.78). The MLP model struggled to predict *Bu. africanus* distribution; here, the model performed reasonably well, showing very good predictive accuracy and model fit for *Bi. pfeifferi*, a species that is rare and patchy in the LSV. This highlights significant variability in performance for the MLP and more stable performance and consistent results for the RF and SVM. Overall, the RF, SVM, and MLP models provided acceptable results, with SVM being the top performer across metrics (Table 3 and Supplementary Fig. 2).

## Conclusions

Two conclusions can be drawn from this study. First, the habitat suitability map for the *Bu. africanus* group and *Bi. pfeifferi* in LSV reveals that the distribution of suitable habitats for potential urogenital and intestinal schistosomiasis transmission is not uniform and typically non-overlapping. The *Bu. africanus* group habitat is the most dominant and abundant, indicating a potential widespread and high risk of urogenital schistosomiasis across the valley. In contrast, environmental conditions, in climatic terms, appear harsher for *Bi. pfeifferi*. Nonetheless, while the *Bi. pfeifferi*-suitable habitat is patchy and scarce, indicating a potential lower risk of intestinal transmission, there is still reason to suggest that the valley has a nonnegligible risk profile. Second, in LSV, vegetation cover is the most important predictor of *Bu. africanus* group distribution, whereas precipitation variables are most important for *Bi. pfeifferi*. This spatial understanding is crucial for current and future targeted snail surveillance and control.

Therefore, this study deduces that the complex interplay between climate change and schistosomiasis ecology emphasizes the need for tailored control measures for both intestinal and urogenital schistosomiasis. For *Bu. africanus*, environmental monitoring in areas predicted to gain suitability is essential, coupled with epidemiological surveys to monitor disease spread. For *Bi. pfeifferi*, the focus should be on sustaining control efforts in the core habitats that are projected to remain suitable, particularly in the agricultural irrigated land of Nchalo Sugar Estate.

## Supplementary Information

Additional file 1.

## Data Availability

Data supporting the main conclusions of this study are included in the manuscript. Historical climate and elevation data are provided by WorldClim and are available at https://www.worldclim.org/data/bioclim.html.

## Abbreviations

SDM: Species distribution model
LSV: Lower Shire Valley
NDVI: Normalized difference vegetation index
ML: Machine learning
RF: Random forest
SVM: Support vector machine
MLP: Multilayer perceptron
VIF: Variance inflation factor
ROC: Receiver operating characteristics
AUC: Area under the curve
TSS: True skill statistic
COR: Correlation
