# Supplementary material for: Predicting current habitat suitability for intermediate snail hosts of urogenital and intestinal schistosomiasis in the Lower Shire Valley floodplain of southern Malawi
Source: Parasit Vectors. 2025 Aug 29;18:368. doi: 10.1186/s13071-025-06952-3 (PMC12395679; doi:10.1186/s13071-025-06952-3)
Supplement: Supplementary file 1 — Additional file 1. [file 13071_2025_6952_MOESM1_ESM.pdf]

Supplementary Table 1

The environmental variables used in this study. For the complete description of each variable, please refer to the data source. Among the 24 input variables, 15 variables were identified with collinearity problems, namely, bio2, bio4, bio5, bio8, bio9, bio10, bio11, bio12, bio16, bio17, bio18, bio19, bio14 and elevation. This indicates that these variables are highly correlated with each other, which can cause inflated uncertainty parameter estimation, unstable variable importance scores and less reliable coefficient estimates in the prediction model.

Variable	Description	Source
bio1	Mean annual air temperature in ℃.	WordClim
bio2	Mean diurnal air temperature range in ℃. That is, the difference between the mean of monthly maximum and minimum temperatures.	WordClim
bio3	Isothermality: The ratio of the diurnal variation to the annual temperature range, expressed as percentage.	WordClim
bio4	Temperature seasonality: The standard deviation of monthly mean or seasonal temperatures.	WordClim
bio5	Maximum temperature of warmest month.	WordClim
bio6	Minimum temperature of coldest month.	WordClim
bio7	Temperature annual range: The difference between the maximum temperature of the warmest month and the minimum temperature of the coldest month (bio5-bio6).	WordClim
bio8	Mean temperature of wettest quarter: The average temperature during the wettest three-month period.	WordClim
bio9	Mean temperature of driest quarter: The average temperature during the driest three-month period.	WordClim
bio10	Mean temperature of warmest quarter: The average temperature during the warmest three-month period.	WordClim
bio11	Mean temperature of coldest quarter: The average temperature during the coldest three-month period.	WordClim
bio12	Annual precipitation: The total precipitation over the course of a year.	WordClim
bio13	Precipitation amount of wettest month: The total precipitation during the wettest month of the year.	WordClim
bio14	Precipitation of driest month: The total precipitation during the driest month of the year.	WordClim
bio15	Precipitation seasonality: The coefficient of variation of monthly or seasonal precipitation.	WordClim
bio16	Precipitation of wettest quarter: The total precipitation during the wettest three-month period.	WordClim

bio17	Precipitation of driest quarter: The total precipitation during the driest three-month period.	WordClim
bio18	Precipitation of warmest quarter: The total precipitation during the warmest three-month period.	WordClim
bio19	Precipitation of coldest quarter: The total precipitation during the coldest three-month period.	WordClim
Elev	Topographic elevation (m).	MASDAP
Slope	Slope (degrees).	WorldPop
Soil	Dominant reference soil groups.	MASDAP
Dist_water	Distance to nearest water body.	WorldPop
NDVI	Mean annual normalized difference vegetation index	Google Earth Engine: Sentinel-2

Supplementary Figure 1.

The nine environmental variables with acceptable levels of multicollinearity included in the predictive model of *Bu. africanus* and *Bi. pfeifferi*.

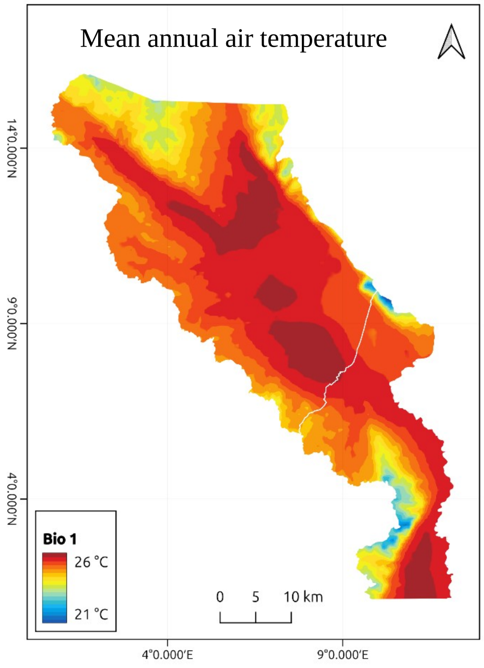

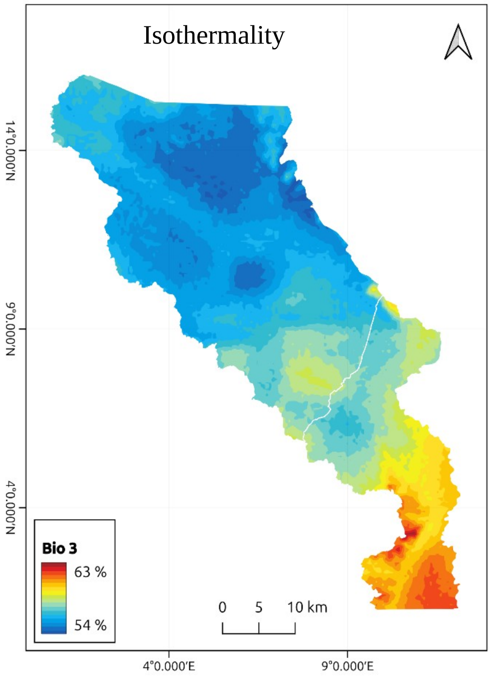

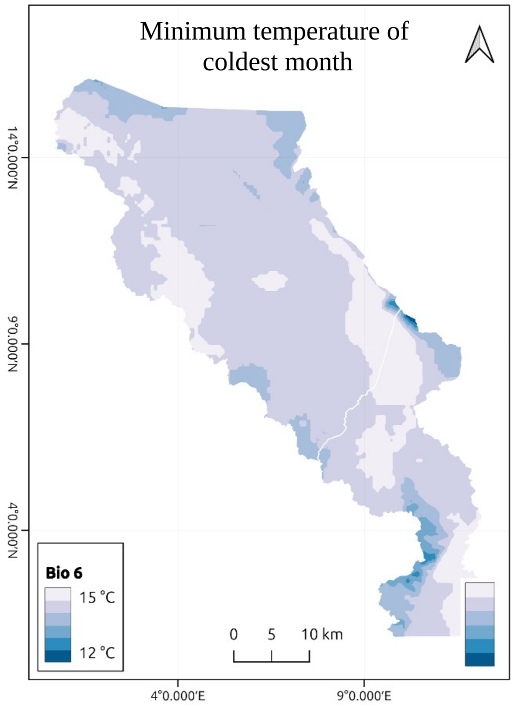
Minimum temperature of coldest month
14°0.000'N
9°0.000'N
4°0.000'N
Bio 6
15 °C
12 °C
0 5 10 km
4°0.000'E
9°0.000'E

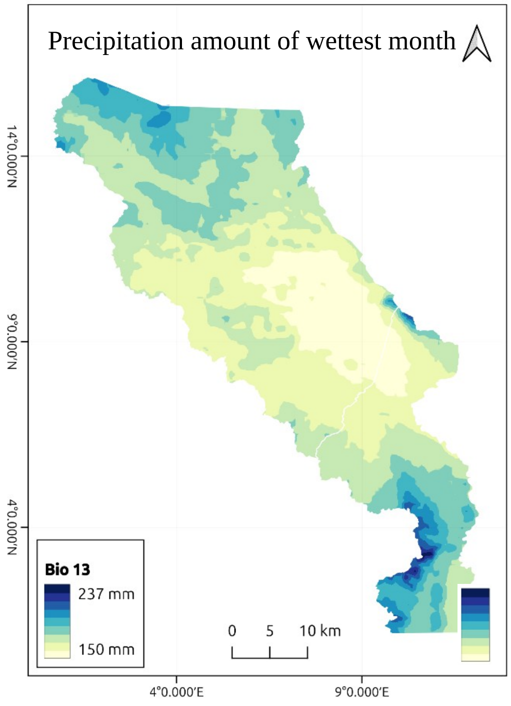
Precipitation amount of wettest month
14°0.000'N
9°0.000'N
4°0.000'N
Bio 13
237 mm
150 mm
0 5 10 km
4°0.000'E
9°0.000'E

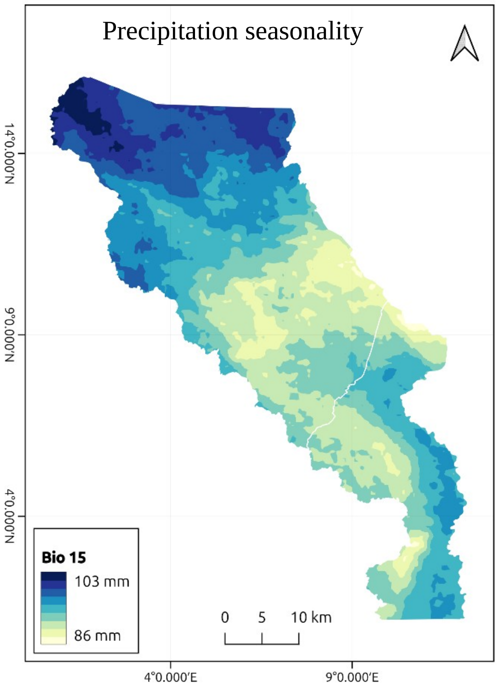
Precipitation seasonality
Bio 15
103 mm
86 mm
0 5 10 km
14°0.000'N
9°0.000'N
4°0.000'N
4°0.000'E
9°0.000'E

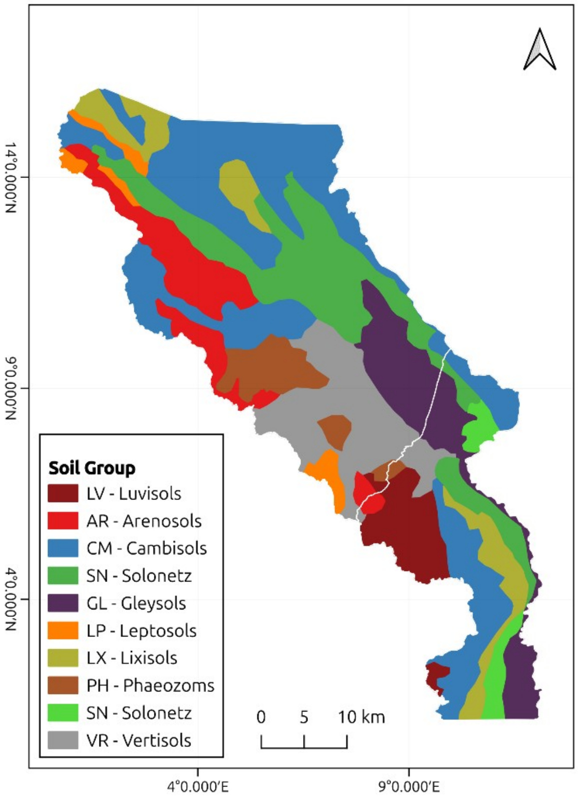
Soil Group
LV - Luvisols
AR - Arenosols
CM - Cambisols
SN - Solonetz
GL - Gleysols
LP - Leptosols
LX - Lixisols
PH - Phaeozoms
SN - Solonetz
VR - Vertisols
0 5 10 km
14°0.000'N
9°0.000'N
4°0.000'N
4°0.000'E
9°0.000'E

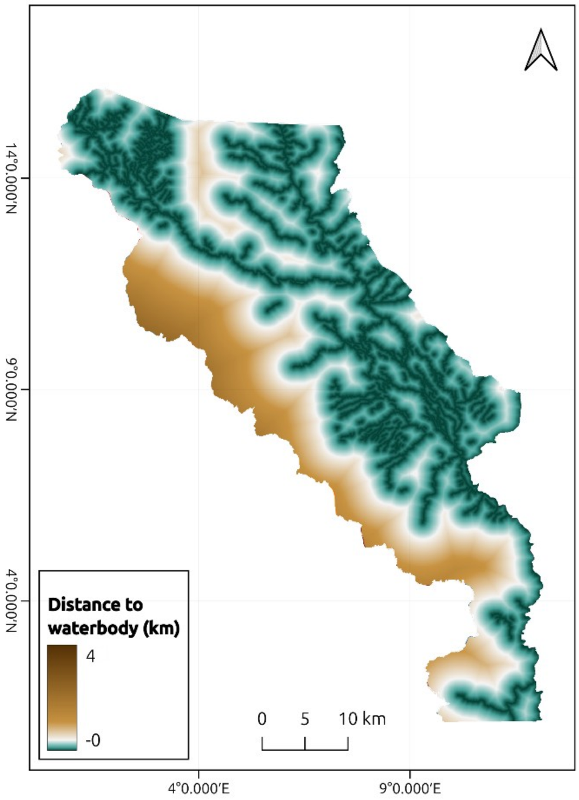
Distance to waterbody (km)
4
-0
0 5 10 km
14°0.000'N
9°0.000'N
4°0.000'N
4°0.000'E
9°0.000'E

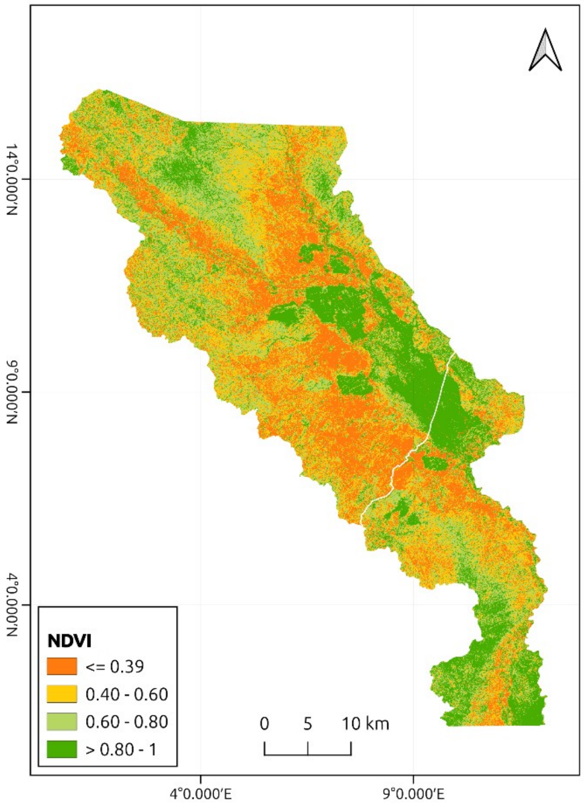
14°0.000'N
9°0.000'N
4°0.000'N
NDVI
<= 0.39
0.40 - 0.60
0.60 - 0.80
> 0.80 - 1
0 5 10 km
4°0.000'E
9°0.000'E

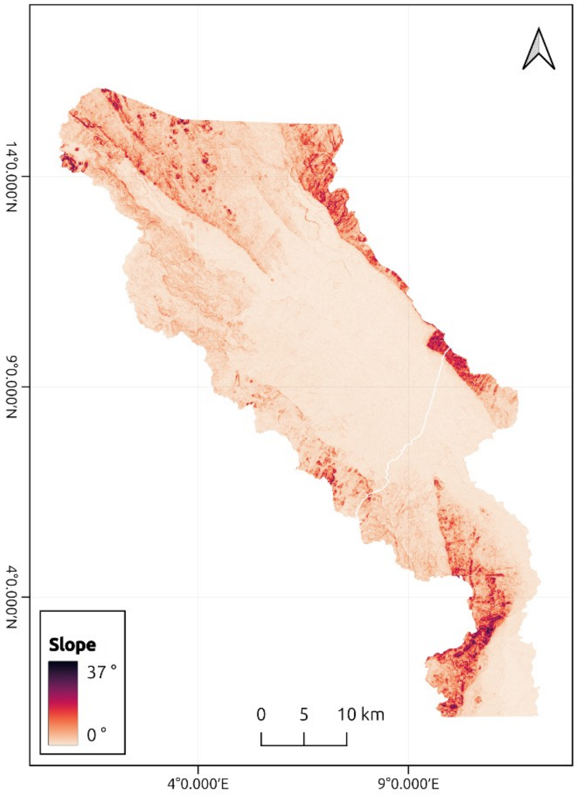
14°0.000'N
9°0.000'N
4°0.000'N
Slope
37 °
0 °
0 5 10 km
4°0.000'E
9°0.000'E

Supplementary Figure 2

ROCs of *Bu. africanus* and *Bi. pfeifferi* predictive models under two resampling techniques: cross-validation and bootstrap. The AUC values varied between the training and test sets, indicative of the differences in the models' capacity to generalize to unseen data. For *Bu. africanus* predictions, the RF model with bootstrap resampling demonstrated better generalization (AUC: 0.981 training, 0.918 test). In contrast, cross-validation showed significantly lower test AUC (0.690) and a high training AUC (0.985), suggesting the possibility of overfitting. Similarly, the SVM model showed better generalization with bootstrap resampling (AUC: 0.872 training, 0.767 test) in comparison to cross-validation (AUC: 0.932 training, 0.736 test). The MLP model also performed well with bootstrap resampling (AUC: 0.912 training, 0.838 test). However, it showed significant overfitting in cross-validation, as evidenced in the test AUC dropping significantly to 0.51, despite a high training AUC of 0.938.

In predicting *Bi. pfeifferi* distribution, the RF model results show satisfactory performance across both resampling techniques, as evidenced by its high AUC scores for both the training and test sets. Specifically, the bootstrap (AUC: 0.984 training, 0.945 test) and cross-validation (AUC: 0.980 training, 0.733 test) scores showed relatively consistent model performance. Similarly, the SVM model demonstrated success in both resampling strategies, achieving an AUC of 0.933 (training) and 0.852 (test) under bootstrapping. This suggests good generalization. The cross-validation results were AUC 0.957 (training) and AUC 0.770 (test), indicative of generally good generalization. The MLP model also achieved high AUC scores of 0.975 (training) and 0.954 (test) under bootstrap evaluation, indicating minimal overfitting. The cross-validation AUC results were 0.953 for training and 0.78 for test, implying a reasonably good training. Taken together, here bootstrap resampling yielded more consistent and reliable results for each model.

Bu. africanus model

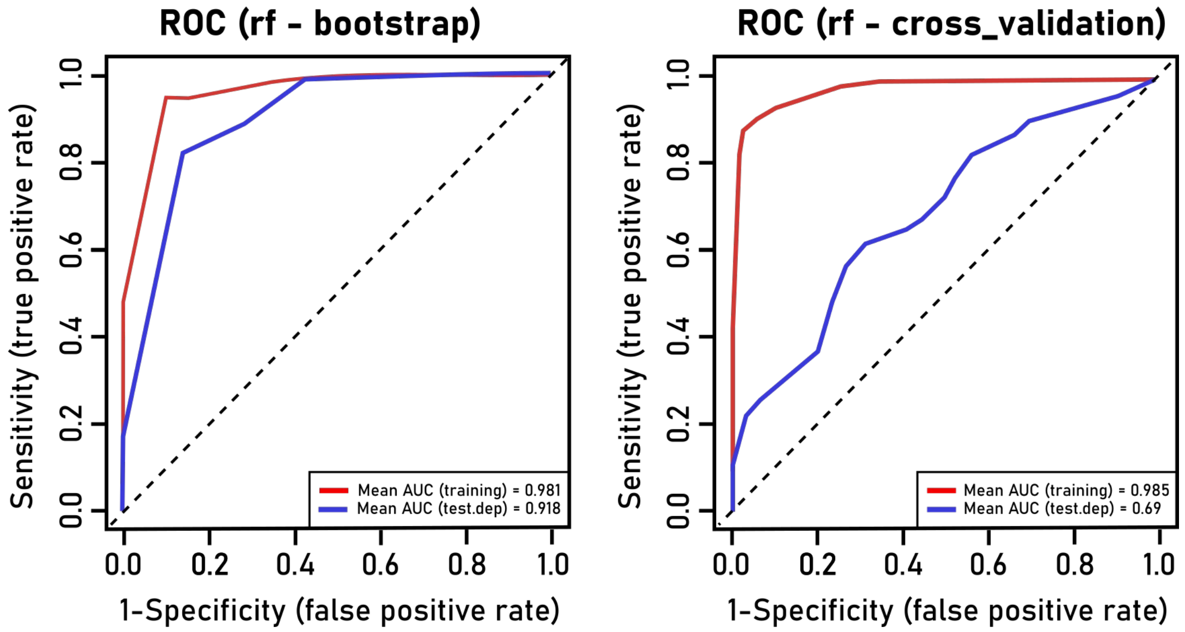

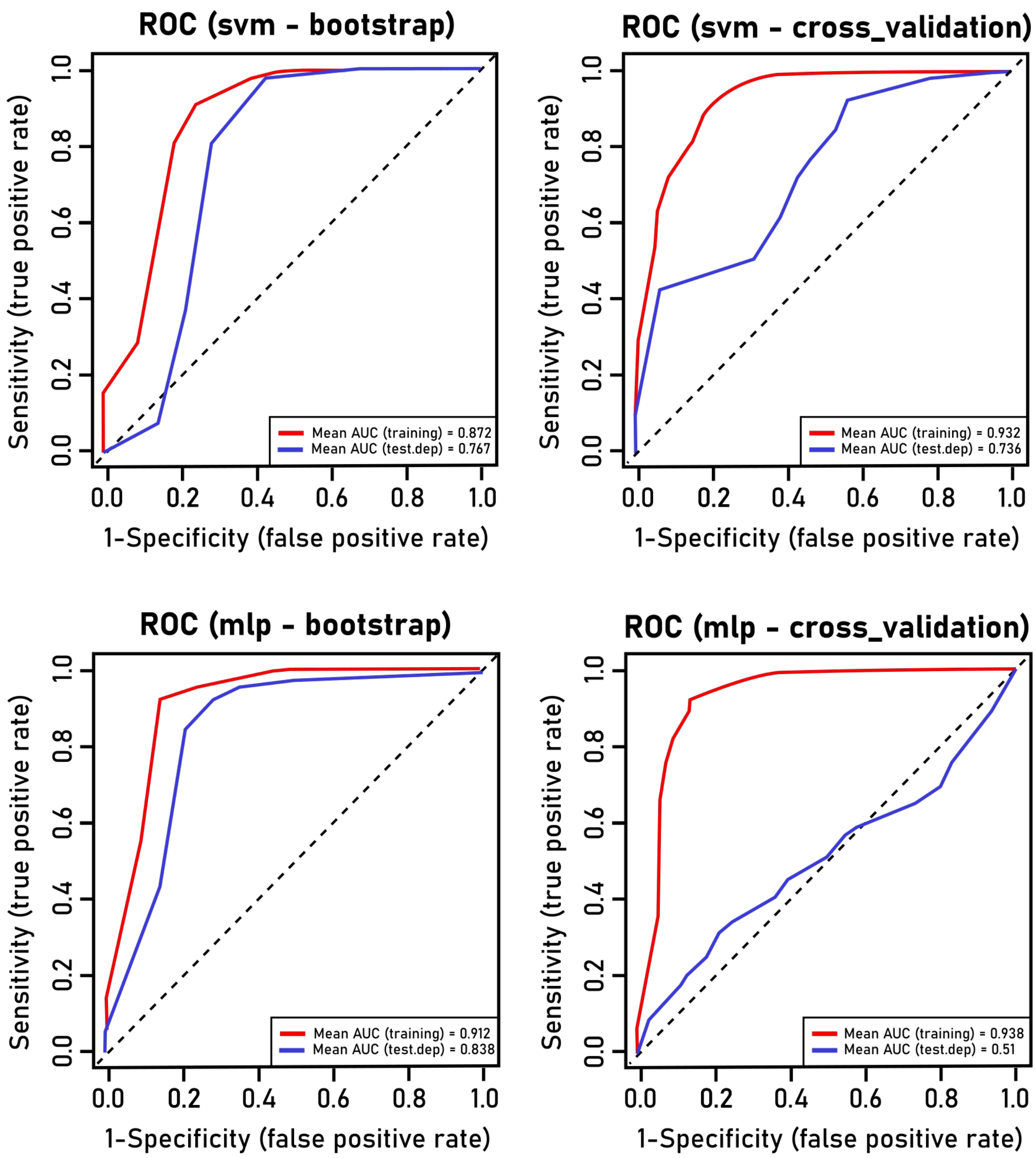

ROC (svm - bootstrap)
Mean AUC (training) = 0.872
Mean AUC (test.dep) = 0.767
ROC (svm - cross_validation)
Mean AUC (training) = 0.932
Mean AUC (test.dep) = 0.736
ROC (mlp - bootstrap)
Mean AUC (training) = 0.912
Mean AUC (test.dep) = 0.838
ROC (mlp - cross_validation)
Mean AUC (training) = 0.938
Mean AUC (test.dep) = 0.51
Sensitivity (true positive rate)
1-Specificity (false positive rate)
0.0
0.2
0.4
0.6
0.8
1.0

Bi. pfeifferi model

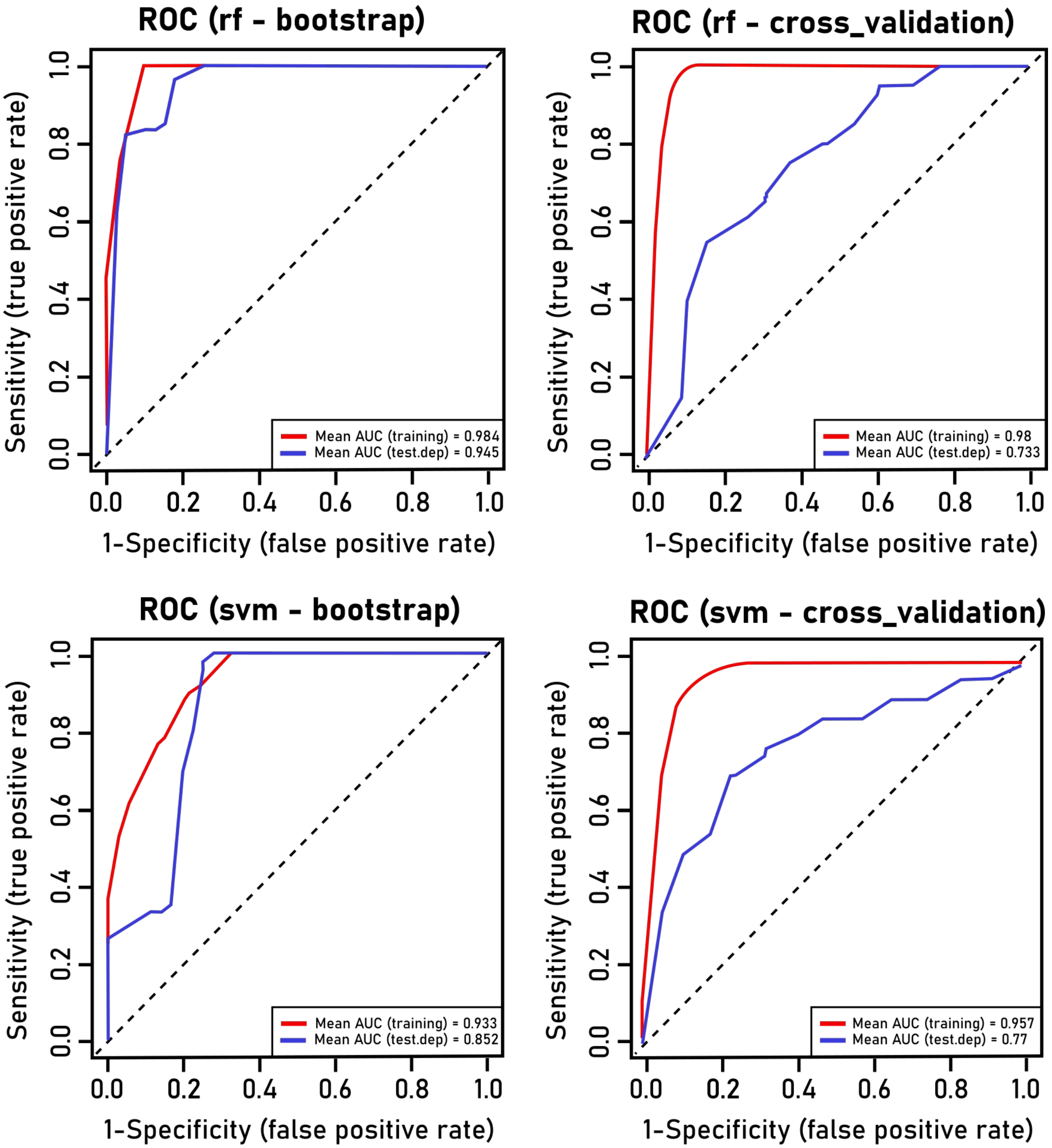

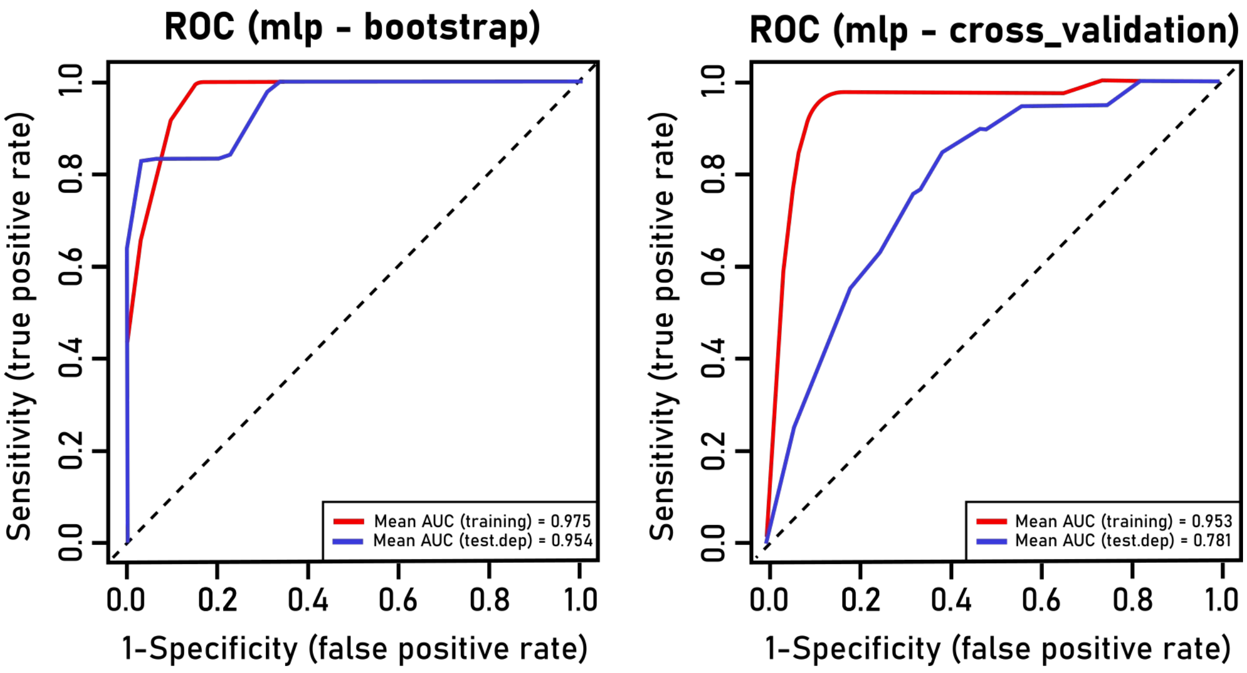

ROC (mlp - bootstrap)
Sensitivity (true positive rate)
1-Specificity (false positive rate)
Mean AUC (training) = 0.975
Mean AUC (test.dep) = 0.954
ROC (mlp - cross_validation)
Sensitivity (true positive rate)
1-Specificity (false positive rate)
Mean AUC (training) = 0.953
Mean AUC (test.dep) = 0.781

Supplementary Figure 3

Current habitat suitability maps for each of the three tested models (SVM, RF and MLP). These maps represent the mean predictions across all replications for each replication method (cross validation and bootstrap).

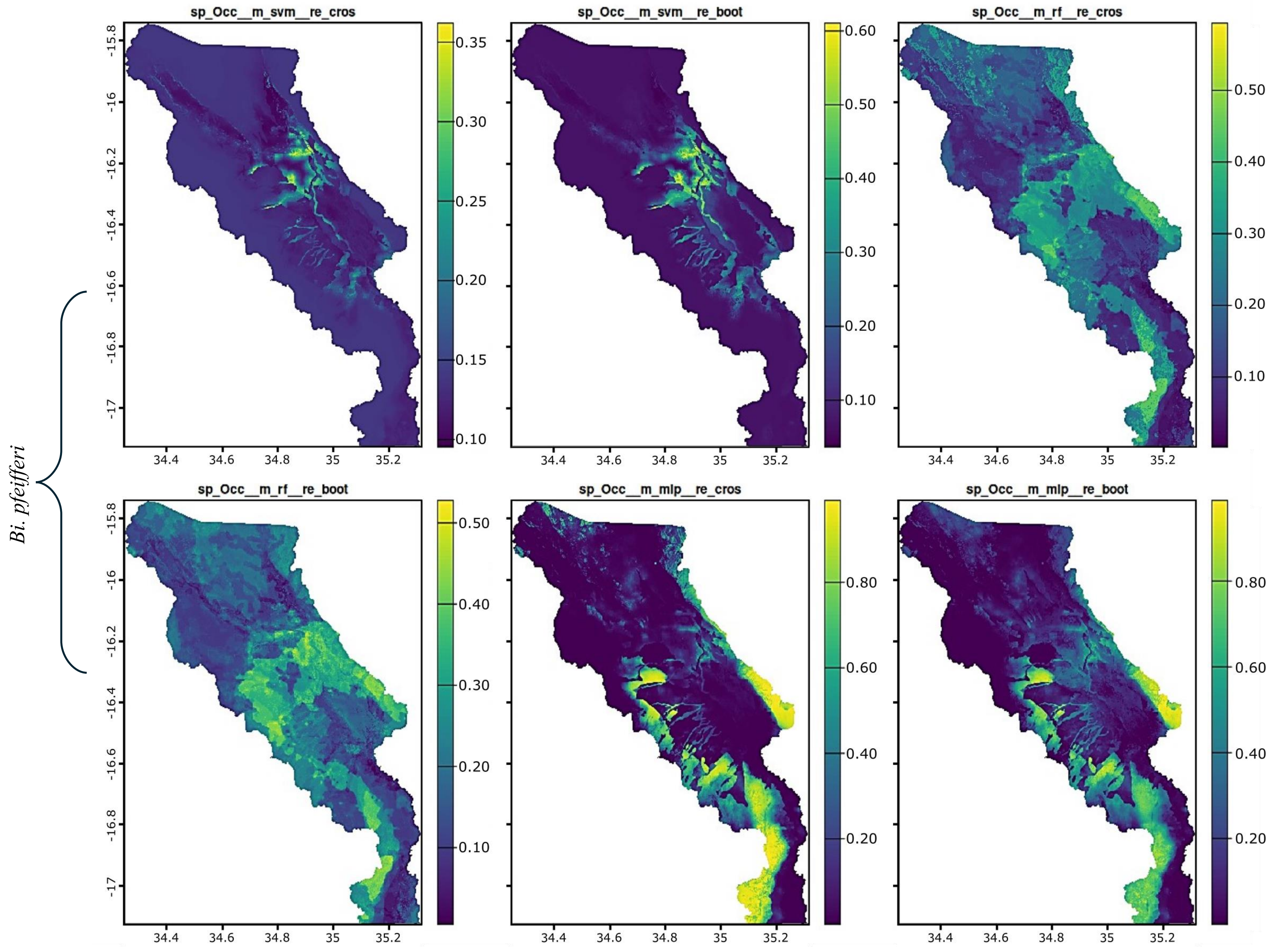

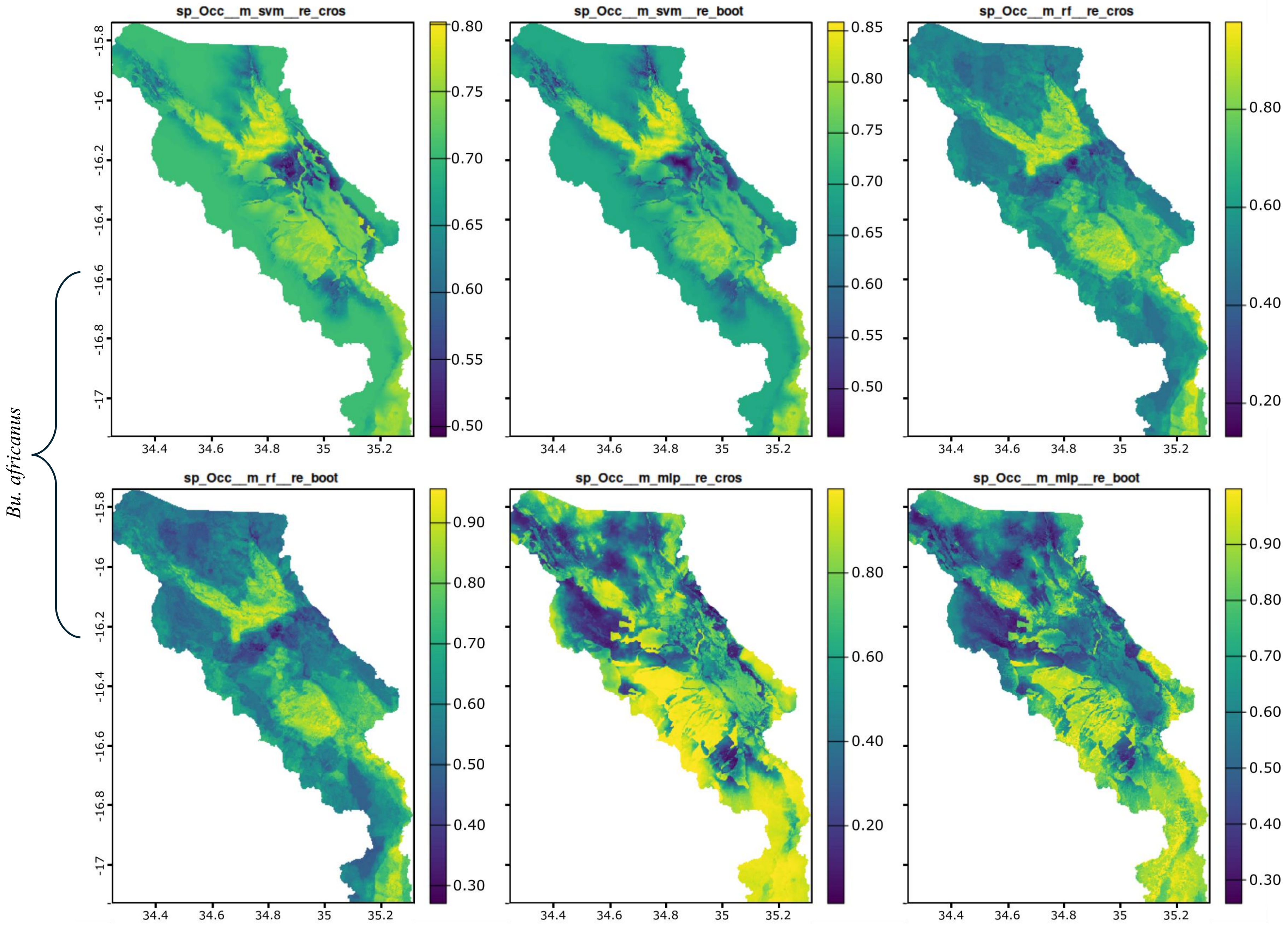

Bu. africanus
sp_Occ__m_svm__re_cros
sp_Occ__m_svm__re_boot
sp_Occ__m_rf__re_cros
sp_Occ__m_rf__re_boot
sp_Occ__m_mlp__re_cros
sp_Occ__m_mlp__re_boot
-15.8
-16
-16.2
-16.4
-16.6
-16.8
-17
34.4
34.6
34.8
35
35.2
